# Legacy of Compassion: The Life and Contributions of Dr. Ronald Joseph Garst in Advancing Orthopaedic Surgery in Bangladesh

**DOI:** 10.7759/cureus.61437

**Published:** 2024-05-31

**Authors:** Md Hasibuzzaman, Kazi Shamim Uzzaman, Dibakar Sarkar, Abdullah Al Mamun Choudhury

**Affiliations:** 1 Department of Trauma and Orthopaedics, National Institute of Traumatology and Orthopaedic Rehabilitation, Dhaka, BGD; 2 Department of Sports Medicine, National Institute of Traumatology and Orthopaedic Rehabilitation, Dhaka, BGD

**Keywords:** history of orthopaedic surgery in bangladesh, orthopaedic care for liberation fighters, medical innovation in bangladesh, trauma care, historical vignette, ortho surgery, rihd, nitor, bangladesh orthopaedic society, dr. ronald joseph garst

## Abstract

Dr. Ronald Joseph Garst, a distinguished spine surgeon and missionary, significantly impacted the field of orthopaedic surgery in Bangladesh, especially during and after the country's Liberation War, when the nation had no orthopaedic specialists. His experiences during Bangladesh's struggle for independence inspired him to establish rehabilitation centers for injured freedom fighters and to found the Rehabilitation Institute and Hospital for the Disabled (RIHD), which later became the National Institute of Traumatology and Orthopaedic Rehabilitation (NITOR), Bangladesh's first tertiary-level trauma center.

In Bangladesh, Dr. Garst was critical in organizing care for injured freedom fighters, setting up a central limb and brace center, and launching a post-graduate training program for orthopaedics, physiotherapists, and occupational therapists. He successfully raised funds, attracted international support, and provided essential training to Bangladeshi doctors, nurses, and limb-makers.

Dr. Garst's legacy extends beyond his medical achievements; his humanitarian spirit and dedication to helping the underprivileged earned him honorary citizenship in Bangladesh. He remained committed to supporting ongoing efforts at RIHD, frequently visiting Bangladesh and contributing equipment and training materials until his passing. Dr. Garst's contributions, such as initiating morning academic sessions at RIHD, continue to influence the orthopaedic community in Bangladesh. This article explores Dr. Garst's remarkable journey, his influence on orthopaedic surgery in Bangladesh, and the enduring impact of his work.

## Introduction and background

In the Annals of Medical History, specific figures stand out for their expertise, skill, and dedication to humanitarian causes. Dr. Ronald Joseph Garst, a pioneering spine surgeon and missionary, is one such figure. His remarkable journey from a small town in Oklahoma to the bustling cities of Bangladesh is a testament to his unwavering commitment to improving orthopaedic care in some of the most challenging environments [1-3].

In this article, we explore the life and legacy of Dr. Ronald Joseph Garst, highlighting his invaluable contributions to the field of orthopaedics in Bangladesh. At the time, Bangladesh was a newly independent nation, emerging from a nine-month war that resulted in millions of deaths and thousands of injuries. There was no orthopaedic surgeon in the country, let alone an orthopaedic hospital, creating a huge humanitarian and health crisis with an enormous burden of injured individuals. Dr. Garst's work was instrumental in addressing this critical need and transforming the country's medical landscape. Born in 1926 in Oklahoma to an agricultural family, Dr. Ronald Joseph Garst graduated from Oklahoma Medical School, where he developed a fascination with orthopaedic surgery. After moving to India, he served for 15 years at the Christian Medical College in Ludhiana, where he honed strong ethics and a passion for serving those in need. When the Liberation War broke out in neighbouring Bangladesh, he reached out to help the injured people of the war-torn nation, pioneering the development of orthopaedic surgery and playing an instrumental role in establishing the Rehabilitation Institute and Hospital for the Disabled (RIHD), later known as the National Institute of Traumatology and Orthopaedic Rehabilitation (NITOR). Dr. Garst's story is one of vision, perseverance, and an unwavering dedication to serving those who need it the most [1-7]. We employed a systematic search strategy across multiple databases, using specific keywords and clearly defined inclusion and exclusion criteria. Additionally, we utilized cross-referencing to ensure comprehensive coverage of Dr. Ronald Joseph Garst's contributions to orthopaedic surgery in Bangladesh. By incorporating recollections from his colleagues and highlighting the ongoing impact of his work, we aim to honour a man whose vision transformed a nation's approach to orthopaedic care.

**Figure 1:**
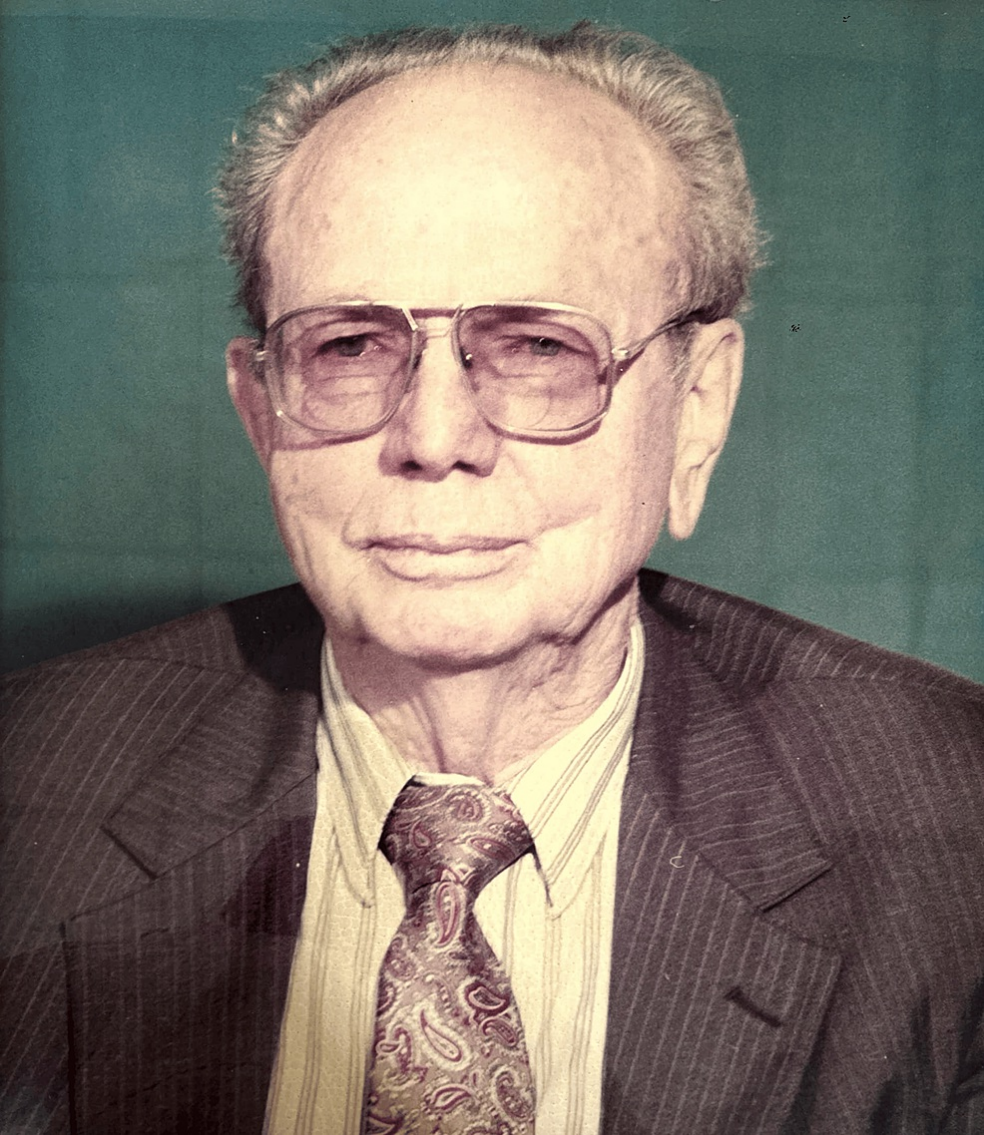
Dr. Ronald Joseph Garst Collected and permission obtained from the Bangladesh Orthopaedic Society

Figure 1 shows Dr. Ronald Joseph Garst.

Table 1 depicts the key events in the life and work of Dr. Ronald Joseph Garst.

**Table 1:** Key events in the life and work of Dr. Ronald Joseph Garst NEC: National Economic Council; RIHD: Rehabilitation Institute and Hospital for the Disabled

Date	Major contribution
1926	Born in Cordell, Oklahoma, USA.
1942	Finished high school at age 16 and began working at Grand Coulee Dam on the Columbia River.
1948	Enrolled at a tuition-free institution in East Tennessee and supported himself through construction and home painting jobs.
Early 1950s	Graduated from Oklahoma Medical School and developed a passion for orthopaedic surgery.
Mid-1950s	Practiced orthopaedics in western Kansas.
1956-1971	Served at Christian Medical College in Ludhiana, India, coordinating orthopaedic services and training for 15 years.
February 1972	Visited Bangladesh with his wife, Marie M. Garst, to assess the problem of war-injured freedom fighters and civilians.
May 1972	Converted the outpatient building of Shaheed Suhrawardy Hospital into the Mukti Bahini Hospital.
January 1973	Recommended Dhaka University Faculty of Medicine to establish a post-graduate course in orthopaedics.
1973-1976	Established a comprehensive orthopaedic program including a 375-bed hospital, prosthetic limb and brace center, and a post-graduate degree in orthopaedic surgery at Dhaka University.
1974	NEC of Bangladesh approved plans for the RIHD and was appointed as project director.
Early 1978	RIHD relocated to a new site, becoming the largest orthopaedic facility in South Asia with 500 beds.
1981	Retired from RIHD and relocated to the USA.
Post-1981-1999	Continued his contributions to orthopaedic surgery and education and received honorary Bangladeshi citizenship in 1999.
1995	Attended the Cincinnati Hand Surgery International Conference in Ohio, USA, along with a delegate of injured freedom fighters who had undergone the Krukenberg hand procedure led by Dr. M. Amjad Hossain.
September 15, 2009	Dr. Garst passed away a couple of months after his wife Marie passed away.

## Review

Early life and education

Born in Cordell, Oklahoma, in 1926, Dr. Ronald Joseph Garst was the son of an agricultural family. His childhood on the family farm instilled a work ethic and ingenuity. Dr. Garst joined the Boy Scouts as a teenager to improve his problem-solving and leadership skills. At 16, he finished high school and got a job at the Grand Coulee Dam on the Columbia River. He worked here in several jobs, including pipe testing [2].

Dr. Garst's father encouraged him to study while working on building projects. After considering this advice, he enrolled at a tuition-free institution in East Tennessee and worked in construction and home painting to support himself. Dr. Garst graduated from college despite personal challenges. After graduating from college, he chose to attend Oklahoma Medical School. This experience sparked his passion for orthopaedic surgery, which changed his life. He worked hard in medical school and acquired a profound interest in human anatomy from his love of building and fixing things [2,3].

After graduating from medical school, Dr. Garst practiced orthopaedics in western Kansas. His career changed when the Kansas United Methodist Church supported his going abroad. This opportunity led him to the Christian Medical College in Ludhiana, India, where he coordinated orthopaedic services and training for 15 years. His overseas experience during his early years shaped his work in Bangladesh, where he had a lasting impact [2,3].

Medical career in Bangladesh

Dr. Garst was a physician at Ludhiana Christian Medical College in India, providing care for the suffering human race in 1971 during the Liberation War of Bangladesh. Seeing the agony endured by the freedom fighters impacted him greatly. He went to the battle camps and started tending to the wounded liberation fighters; many of them were unable to walk due to their injuries. Justice Abu Sayeed Chowdhury, the Government of Bangladesh's exile's special envoy at the time, became aware of his admirable deed [2,3].

Meanwhile, Bangladesh gained its independence, and Justice Chowdhury asked Dr. Garst to visit Bangladesh. On February 28, 1972, Garst and his wife, Marie M. Garst, traveled to Bangladesh because of their compassion for freedom fighters who had been injured in battle. With the government's support and the wishes of the father of the nation Bangabandhu Sheikh Mujibur Rahman, he established temporary rehabilitation centers for injured liberation fighters at Mohammadpur College Gate, which is close to Shaheed Suhrawardy Hospital in Sher-e-Bangla Nagar [1-4].

**Figure 2:**
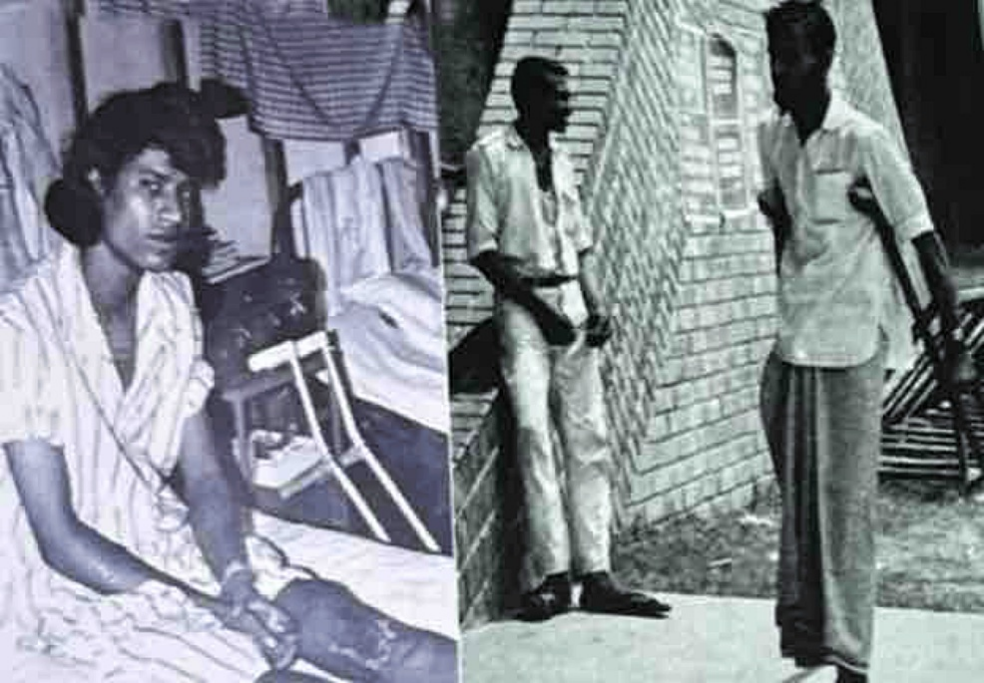
Freedom fighter taking service from the RIHD now NITOR RIHD: Rehabilitation Institute and Hospital for the Disabled; NITOR: National Institute of Traumatology and Orthopaedic Rehabilitation Collected and used with permission from the Bangladesh Orthopaedic Society

After the glorious Liberation War, the injured and victims of the Liberation War were flooding the nation's hospitals. Dr. and Mrs. Garst made every attempt to find a solution after witnessing the victims' immense suffering. They met with the Secretary, the then-minister, and other top officials of the Ministry of Health, who asked him to establish a central limb and brace center and orthopaedic hospital nationwide to care for all the veterans of the liberation wars [2,3]. Dr. Garst began his fund-raising campaign right once and briefed the friendly nations about the requirements of the newly formed nation. Several foreign nations and other organizations made kind offers in response to his appeal. He managed to turn the outpatient department of Shaheed Suhrawardy Hospital into a hospital with state-of-the-art OT facilities in less than a month, where injured freedom fighters came for treatment of their injured limb (Figure 2). There were only 100 beds, not nearly enough to handle the influx of patients. The number of beds was increased to 150 at the end of the second month. In order to determine the state of the injured patients, Dr. Garst began visiting district and cantonment hospitals around the nation. He then set up care facilities for the patients at this hospital [2,3].

"Dr. Garst, you equip and start a full-fledged Orthopaedic Hospital and Limb Center and run them for one year, the Government of Bangladesh will be able to carry it on with the project," said Dr. Taijul Hossain, the then-secretary of health and family planning for the Government of Bangladesh, to Dr. Garst. This served as a great source of motivation for him and his devoted team [2].

The hospital opened to general patients with orthopaedic and trauma problems after the majority of the war casualties finished their treatment in eight months. At that time, the hospital building's converted roof was used to provide an additional 100 beds for women and children. A center for prosthetic limbs and braces was operational within a year, and a large number of prosthetic limbs were distributed to freedom fighters and casualties of the Liberation War. The Government of the People's Republic of Bangladesh assumed full responsibility for the hospital's operations on July 1, 1973, which had grown to 250 beds [5,6].

At that time, it became clear that a separate orthopaedic and traumatology hospital with appropriately qualified physicians and specialists was necessary to satisfy the overwhelming demand for care of the physically challenged person in the country. So, a strategy was created to satisfy all conditions set forth by the Overseas Development Administration of England, with the full cooperation of leading surgeons across the nation and the Ministry of Health. The authorities approved the plan on schedule. Then, under the direction of England's Overseas Development Administration, foreign orthopaedic doctors from various countries, including the UK, USA, Canada, Australia, and India, were attracted to Bangladesh to contribute to the advancement of orthopaedics in the country. These surgeons included Mr. J.N. Wilson, Sir Pulvertaft, Mr. Tricky, Mr. Powell, Mr. Eyre Brook, Mr. Gibson, and Mr. Walker from the UK; Mr. Ditmanson, Mr. Conner, Mr. Jack Both, and Mr. Poul Spray from the USA; Mr. Hubert, Mr. Hasan, and Mr. Sharif from Canada; Mr. Walsh from Australia; and Mr. Bazliel and Mr. Sood from India [3].

Establishing post-graduate training and physiotherapy program

Recognizing the gravity of the dire need to serve people after arriving in Bangladesh, Dr. Garst requested Bangabandhu Sheikh Mujibur Rahman to send some Bangladeshi doctors abroad for training in orthopaedic surgery. Following this request, Bangabandhu directed that in May 1972, five doctors (Dr. Mohammad Shamsuddin Ahmed, Dr. Ruhul Amin, Dr. Badruddoza, Dr. K. M. Alam, and Dr. Momin Ullah), 17 nurses, and 12 limb-makers be sent to East Germany for training. It's worth noting that, at that time, Bangladesh had not yet issued passports, so these individuals traveled using a "travel document" handwritten by Bangabandhu, with a limited amount of dollars. Upon their return, they dedicated themselves to serving wounded freedom fighters and other underprivileged patients in the country [5,6].

Dr. Garst recommended starting a post-graduate program in orthopaedic surgery in Bangladesh, which was subsequently initiated at the University of Dhaka. In July 1973, the first post-graduate program in Bangladesh, which included an MS and a Diploma in Orthopaedic Surgery and a BSc in Physiotherapy, was launched. By 1976, a total of nine doctors had completed their orthopaedic training, including five who earned their MS degrees and four who obtained their D Orth degrees, marking the beginning of a new era of orthopaedic specialists. Three occupational therapists and 12 physiotherapists received their BSc degrees. Twelve recently graduated nurses finished a year-long orthopaedic nursing program. A three-year course in the production of prosthetic limbs and braces has taught 26 young men. As a result, 1976 was Bangladesh's most incredible year for orthopaedic surgery to date [1-3,5]. In addition, together with the Indian plastic surgeon Dr. P. Buzwel, Dr. Garst set up the first unit of plastic and reconstructive surgery in Bangladesh. In this sense, Dr. Garst was instrumental in the development of orthopaedic surgery, plastic surgery, and physiotherapy in war-torn Bangladesh [7].

**Figure 3:**
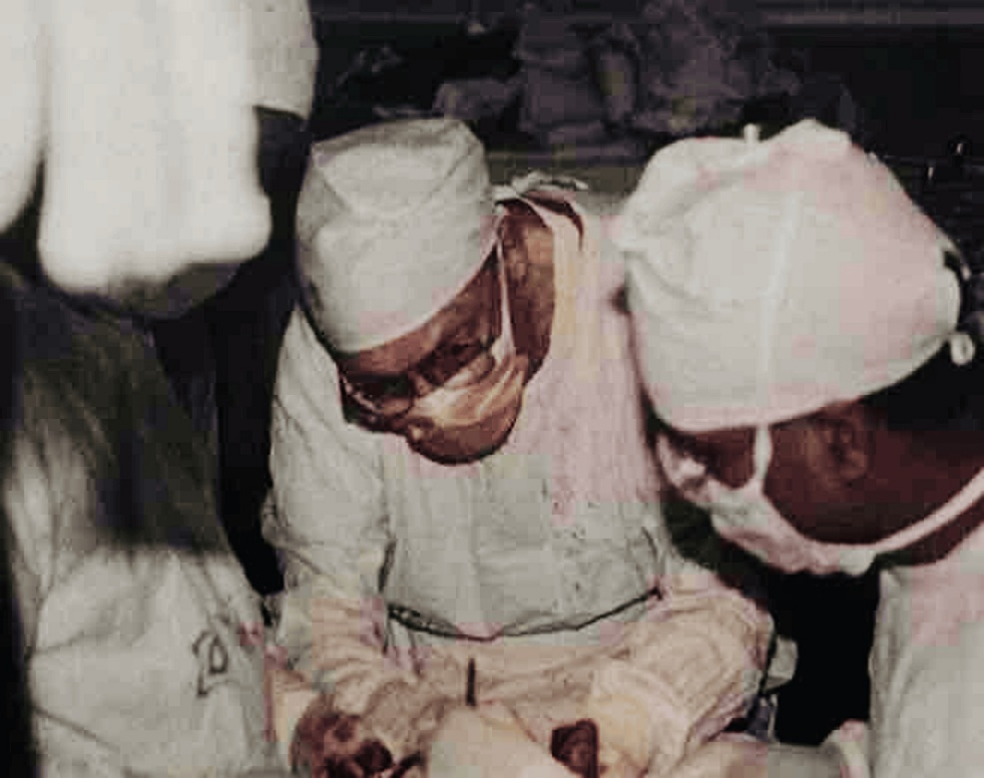
Dr. Garst in the operating theatre of the RIHD now NITOR RIHD: Rehabilitation Institute and Hospital for the Disabled; NITOR: National Institute of Traumatology and Orthopaedic Rehabilitation Collected and used with permission from the Bangladesh Orthopaedic Society

The casualty ward had been expanded to include 75 beds, bringing the total capacity to 375 at the time this happened. The overall programmed activities ran in a two-storied outpatient department building, including its roof, which was clearly insufficient for a vast population. When patients who were women and children were admitted, more space had to be added, and everyone was forced to consider a regular hospital. Dr. Garst was appointed project director of the projected hospital complex to be constructed at Sher-e-Bangla Nagar, Dhaka, the future capital of Bangladesh, after the National Economic Council of Bangladesh approved the designs of RIHD in 1974. Initially, this facility was known as the Shaheed Suhrawardy Hospital Complex, and Dr. Garst agreed to receive a salary of just 2 dollars in recognition of his services as he continued his service as both surgeon and project director (Figure 3) [2,3,5].

He assembled a large collection of orthopaedic literature published since 1950 for the then-RIHD library, which Marie M. Garst oversaw. This included Dr. Garst's published articles on topics ranging from tuberculosis to Krukenberg hand and poliomyelitis. Today, the library of NITOR was named after Mrs. Marie M. Garst to recognize her contribution to the orthopaedic surgeons of Bangladesh. Dr. Garst always had a typewriter so he could type down whatever needed to be typed and avoid the needless inconvenience of causing delays in formal government procedures [2,3,7,8].

Legacy and impact

**Figure 4:**
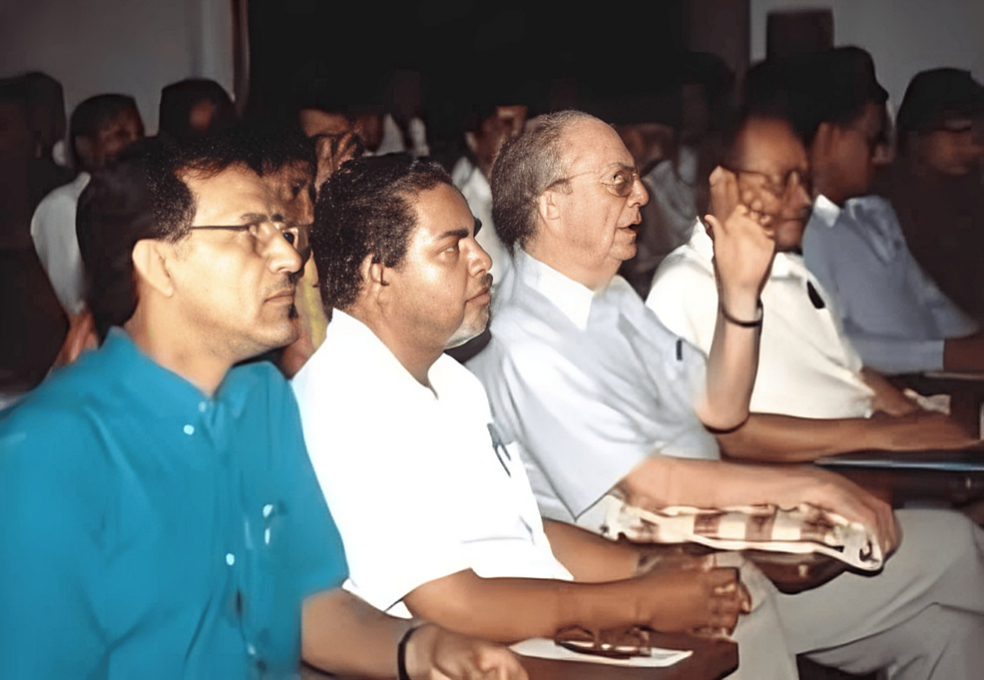
Dr. Garst is participating in the daily academic session in NITOR NITOR: National Institute of Traumatology and Orthopaedic Rehabilitation Collected and used with permission from the Bangladesh Orthopaedic Society

The impact that Dr. Garst has had on the development of orthopaedic surgery was immense. His arrival in our nation raised the bar for orthopaedic surgery by bringing in many internationally recognized surgeons from Singapore, Hong Kong, America, Britain, and Canada. Doctors from our nation were brought to Singapore by Dr. Kunda Pillay of Mount Elizabeth Hospital, where they received orthopaedic training. He provided six months of fellowship training to doctors from Bangladesh. Dr. Garst initiated the morning discussion session at RIHD, which has continued in NITOR. This academic gathering aimed to discuss the work and experiences of the previous day, which was attended by all of the institute's doctors (Figure 4) [1-3,5].

After returning to the USA, Dr. Garst launched a significant initiative by inviting war-injured hand amputees who had undergone the Krukenberg hand procedure, allowing them to perform daily activities without artificial limbs, to the Cincinnati Hand Surgery International Conference in Ohio, USA, likely in 1995. Due to limited finances, he nominated Dr. M. Amjad Hossain, currently the Chief Consultant of the Department of Orthopedic Surgery and Arthroplasty Centre at Labaid Hospital, to lead the Bangladesh delegation. Dr. M. Amjad Hossain coordinated with veteran freedom fighters from the Bangladesh Army to organize the Dhaka-New York flight tickets. In preparation for the conference, TV personality Professor Momtaz Uddin produced a 10-minute documentary titled "War of Bangladesh Independence and Krukenberg Hand" funded largely by Anisul Huq, the then-future Dhaka North City Corporation Mayor. The video was displayed at the conference and received high praise from renowned hand surgeons like Dr. Paul Brand and Dr. Swanson. Approximately 200 copies of the video were distributed at the venue. This initiative highlighted Dr. Garst's commitment to advancing orthopaedic care and his outstanding achievements in the field [9,10].

**Figure 5:**
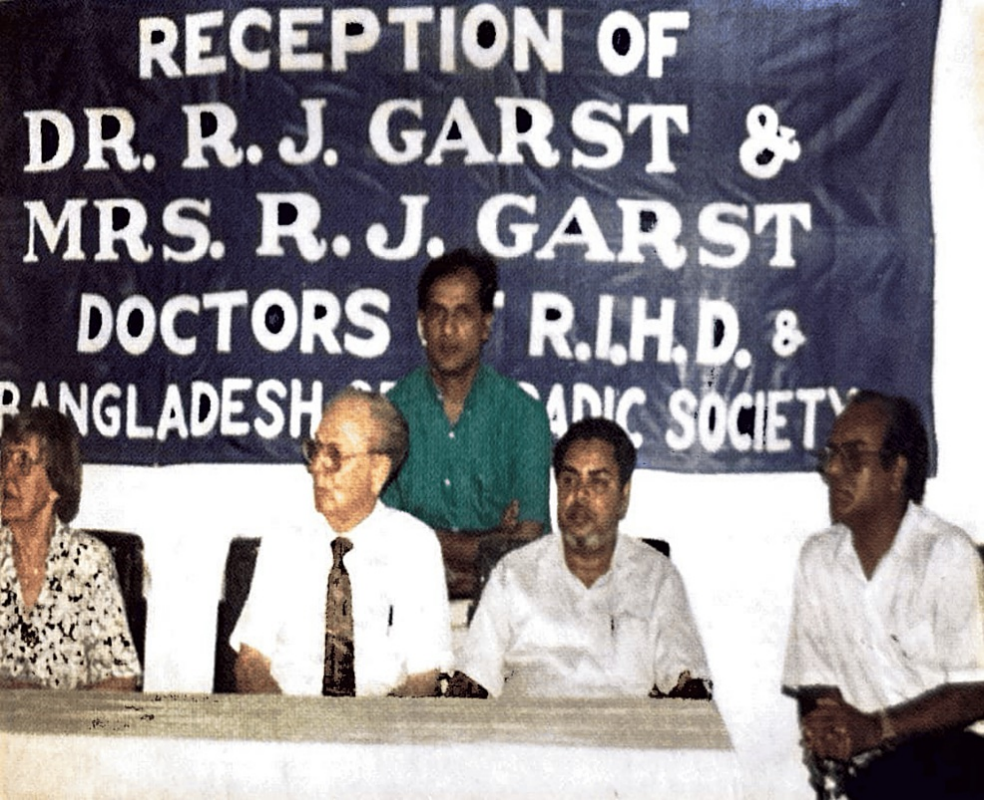
Reception of Dr. Garst and Marie M. Garst by the Bangladesh Orthopaedic Society Collected and used with permission from the Bangladesh Orthopaedic Society

Dr. Garst always focused on serving the impoverished. "God has sent me on a mission to help the suffering humanity of my community and the other communities of the world," which was written in his notepad. Dr. and Mrs. Garst are always warmly welcomed at all events hosted by the Bangladesh Orthopaedic Society in recognition of their significant dedication and contributions to Bangladesh and its orthopaedic community (Figure 5) [1-3].

## Conclusions

Dr. Garst's impact in Bangladesh is characterized by medical ingenuity, a profound sense of empathy, and dedication to helping the disadvantaged. Recognizing his selfless commitment and innovative mindset, he was granted the status of honorary Bangladeshi citizen in 1999. Despite his return to Tennessee in 1981, Dr. Garst maintained regular visits to Bangladesh, providing equipment and training materials to support the ongoing efforts at RIHD until he died in 2009.
